# Phylogeography of the *Bradyrhizobium* spp. Associated With Peanut, *Arachis hypogaea*: Fellow Travelers or New Associations?

**DOI:** 10.3389/fmicb.2019.02041

**Published:** 2019-09-04

**Authors:** Besma Bouznif, Ibtissem Guefrachi, Ricardo C. Rodríguez de la Vega, Mariangela Hungria, Mohamed Mars, Benoit Alunni, Jacqui Anne Shykoff

**Affiliations:** ^1^Écologie, Systématique et Évolution, CNRS, University Paris-Sud, AgroParisTech, Université Paris-Saclay, Orsay, France; ^2^Institute for Integrative Biology of the Cell, UMR 9198, CNRS/Université Paris-Sud/CEA, Gif-sur-Yvette, France; ^3^Research Unit Biodiversity and Valorization of Arid Areas Bioressources (BVBAA), Faculty of Sciences, Gabès, Tunisia; ^4^Embrapa Soja, Londrina, Brazil

**Keywords:** legume-rhizobium association, symbiosis, horizontal gene transfer, host range, novel host-symbiont associations

## Abstract

Legume plants have colonized almost all terrestrial biotopes. Their ecological success is partly due to the selective advantage provided by their symbiotic association with nitrogen-fixing bacteria called rhizobia, which allow legumes to thrive on marginal lands and nitrogen depleted soils where non-symbiotic plants cannot grow. Additionally, their symbiotic capacities result in a high protein content in their aerial parts and seeds. This interesting nutritional value has led to the domestication and agricultural exploitation of several legumes grown for seeds and/or fodder for human and domestic animal consumption. Several cultivated legume species are thus grown far beyond their natural geographic range. Other legume species have become invasives, spreading into new habitats. The cultivation and establishment of legume species outside of their original range requires either that they are introduced or cultivated along with their original symbiotic partner or that they find an efficient symbiotic partner in their introduced habitat. The peanut, *Arachis hypogaea*, a native of South America, is now cultivated throughout the world. This species forms root nodules with *Bradyrhizobium*, but it is unclear whether these came with the seeds from their native range or were acquired locally. Here we propose to investigate the phylogeography of *Bradyrhizobium* spp. associated with a number of different wild and cultivated legume species from a range of geographical areas, including numerous strains isolated from peanut roots across the areas of peanut cultivation. This will allow us to address the question of whether introduced/cultivated peanuts associate with bacteria from their original geographic range, i.e., were introduced together with their original bacterial symbionts, or whether they acquired their current associations *de novo* from the bacterial community within the area of introduction. We will base the phylogenetic analysis on sequence data from both housekeeping and core genes and a symbiotic gene (*nif*). Differences between the phylogenetic signal of symbiotic and non-symbiotic genes could result from horizontal transfer of symbiosis capacity. Thus this study will also allow us to elucidate the processes by which this symbiotic association has evolved within this group of *Bradyrhizobium* spp.

## Introduction

The symbiosis between legume plants and nitrogen-fixing bacteria called rhizobia is a major ecological process in the nitrogen biogeochemical cycle. This symbiosis allows legume plants to colonize N-limited environments, to accumulate large amounts of protein in their seeds and aerial parts and to enrich the soil with an input of fixed nitrogen at the end of their life cycle. Legume plants, with these very useful characteristics, were among the first domesticated plants at the dawn of agriculture, constituting, together with cereals (such as barley, emmer, and einkorn wheats) and flax (the first fiber crop), the so-called Neolithic founder crops. Lentil, chickpea, pea, and bitter vetch were domesticated and have been cultivated for food and feed and selected for high protein content for about 9,500–10,000 years (Zohary and Hopf, 1973). Throughout human history several legumes including pulses, forage crops and trees have been spread all over the world, often intentionally introduced to areas far from their native range. In these new environments, legumes need symbiotic partners to establish a functional symbiosis. If the habitual partners are not introduced along with the host plants nodulating bacteria must be acquired locally from the soil bacterial community in the area of introduction.

The availability of appropriate partners will depend on the biogeography of soil microbes. Are all taxa, potentially, everywhere, but their presence and abundance locally determined by environmental conditions, as was suggested by Baas-Becking in 1934 (de Wit and Bouvier, 2006) with the famous “Everything is everywhere, but the environment selects”? Indeed, a broad survey of the bacterial communities from over 2000 soil samples across France revealed that presence and abundance of most phyla and genera was best explained by environmental drivers such as land use and soil properties (Karimi et al., 2018). However, for four phyla, including beta-proteobacteria that include some rhizobia, spatial parameters explained most of the variation, implying limited dispersal. Thus, a biogeographical signal can be expected in the distribution and abundance of some soil bacteria such that introduced host plants may not find their habitual bacteria at a site of introduction.

Furthermore, at the level below genus, several observations belie the absence of biogeography. First, for pathogenic organisms, quarantine often works, so clearly these pathogenic strains are not everywhere simply waiting for an appropriate host. Secondly for mutualists, several symbiotic legume crops required inoculation, at least initially, to grow well when they were first planted in new areas (see below). Indeed, the introduction success of a symbiotic legume in a new habitat depends on its capacity to find a local bacterial nodulator, and thus symbiosis may represent a barrier to the establishment of symbiotic legumes in a new region (Parker, 2001; Simonsen et al., 2017). To our knowledge there is no general overview of how commonly introduced symbiotic legume species acquire local bacteria at the site of introduction versus how often their symbiotic partner is also introduced. In this paper, we compile information available in the literature about the bacteria that nodulate some major legume crops and trees. Comparing bacterial symbionts from the native and introduced ranges of these host plants allows inference as to whether these plants were introduced with their original symbionts or whether they acquired local bacteria from the community of bacteria nodulating indigenous legume plants. Finally, we focus on peanut (*Arachis hypogaea*), the second pulse and oilseed legume crop after soybean in terms of global production. Peanut is native to the Americas, although the main production areas today are Asia and Africa, making it an interesting case to study the history of legume introduction and nodulation outside the native range.

**FIGURE 1:**
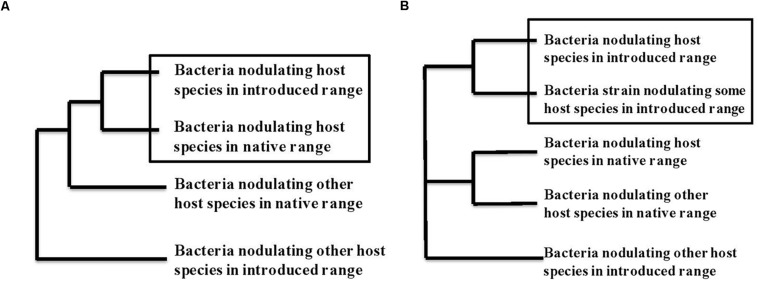
Phylogenetic trace of co-introduction **(A)** versus acquisition of a new symbiotic partner **(B)** in a new geographical zone. **(A)** Depicts scenario 1: If the bacteria found associated with a host species in a new geographic area arrived via co-introduction with the host plant these bacteria should be genetically most similar to the bacteria from the same host plant in its original geographical range. In contrast in **(B)**, scenario 2, if hosts acquire new symbionts from the soil bacterial community in their new geographic range, we expect the strains isolated from the introduced host species to most closely resemble bacterial strains associated with one or more host species within the introduced range. We assume some geographic pattern to relationships of the bacteria, with species from the same geographic range showing more similarity.

Do introduced legumes bring their partners with them or associate with new partners? When rhizobial-associated plants are transported to new environments, they either are introduced together with their nodulating bacteria or they find and recruit mutualistic bacteria from the local rhizobial community. Identifying which process has occurred involves comparing strains of bacteria that nodulate the same plant species in its native and introduced range with bacteria that nodulate other legume species from both ranges. Indeed, if plants are introduced with their habitual nodulating bacteria, the bacterial strains from the native and introduced ranges should cluster together on a tree of genetic similarity, as depicted in Figure 1A. On the other hand, for plants that acquire new rhizobial partners in the introduced area, their bacterial strains from the introduced area should cluster together with bacterial strains from other species of host plant in that same introduced area, with clusters presenting geographical areas and a clear biogeographical pattern (Figure 1B). Several researchers have addressed this question directly, and several studies on the genetic diversity of rhizobial bacteria provide data from which these patterns can be deduced. Here we discuss what we could find for a number of introduced, invasive or cultivated legume species that grow in several different regions of the world.

Some nomadic legumes bring their symbionts with them. Several cultivated species are or were systematically inoculated when planted in new areas. Lupins (*Lupinus* spp.) and *Serradella* (*Ornithopus* spp.) were introduced as forage crops in Australia and South Africa but their successful cultivation required inoculation with co-introduced rhizobia for several decades. Now these plants can grow in both areas without inoculation, but the bacteria that nodulate them are closely related to strains from southern Europe, part of the native range of these forage species (Stȩpkowski et al., 2005), suggesting that the current population of nodulating bacteria stem from the co-introduced inoculum. Similarly, in New Zealand, introduced and invasive *Acacia*, *Cytisus* and *Ulex* species associate with *Bradyrhizobium* microbial partners that are very different from the *Sinorhizobium* that nodulate native species in New Zealand (Weir et al., 2004), while *Dipogon lignosus*, an invasive from South Africa, is nodulated by *Burkholderia* that closely resemble South African strains. Australian *Acacia longifolia* and *A. melanoxylon*, invasive in coastal sand dune habitats of Portugal, are nodulated by their habitual Australian rhizobia that must have been co-introduced with them. Furthermore, these introduced bacteria also can nodulate native Portuguese *Cytisus* and *Ulex* but these native plants derive far less benefit from the foreign bacteria than they do their habitual native bacteria (Rodríguez-Echeverría et al., 2012).

These patterns indicate that several introduced species arrived together with their bacterial symbionts, with which they continued to associate. Indeed, the fact that successful introduction and cropping of some legume species required the concomitant introduction and inoculation of their rhizobial symbiotes suggests that recruiting local bacteria is not always easy. In addition, novel associations between host plant and bacteria may be less efficient than old, coevolved associations (Rodríguez-Echeverría et al., 2012), so even if new partners were available they may provide less advantage than co-introduced, habitual mutualists.

Not surprisingly then, there are fewer examples of plants that, in their introduced range, are nodulated only by novel bacteria that do not resemble the bacteria from their native range. *Mimosa pigra*, a native of the neotropics, presents a mixed picture. This plant is invasive in many areas including Taiwan and Australia. In both invasive areas and in the native range it is nodulated by bacteria of the genus *Burkholderia*. Whereas the bacteria that nodulate this plant in Taiwan closely resemble one of the dominant strains that nodulates this plant in its native range (Chen et al., 2005), the strains in Australia represent several divergent lineages that are unrelated to the native strains. Thus it appears that *M. pigra* was cointroduced with a nodulating strain to Taiwan (Chen et al., 2005) but was not accompanied by its habitual *Burkholderia* on colonizing Australia, where it acquired novel symbiotic *Burkholderia* (Parker et al., 2007).

Several other species associate both with strains that appears to have been co-introduced, being very similar to strains found in the native range of the plant, and have adopted additional new symbiotic partners at the area of introduction. Within introduced populations or areas of cultivation this can be observed by an increase in the genetic diversity of symbiotic bacteria between the native and introduced range. *Acacia pycnantha*, an Australian invader of South Africa and *Robinia pseudoacacia*, a North American native that has been introduced throughout the world and is deemed invasive in some areas, both show a higher diversity of nodulating bacteria in the introduced than the native range. For both plant species bacteria characteristic of the native range were recovered also in the invaded range, together with additional bacterial taxa that represented additional, new partners from the native bacterial communities in the areas of introduction (Chen et al., 2000; Ulrich and Zaspel, 2000; Wei et al., 2009; Tang et al., 2012; Ndlovu et al., 2013).

In addition, horizontal exchange of the genes involved in the symbiotic process can also occur during the introduction of legumes to new habitats. Thus the two possibilities discussed above, of introduced, cultivated or invasive legume species either arriving accompanied by their rhizobial mutualists or acquiring novel ones from the microbial communities in their new geographic range do not cover all the observations of the rhizobia associated with legumes far from their native ranges. At the large evolutionary scale of bacterial genera, symbiotic and non-symbiotic genes in rhizobial bacteria appear to have distinct evolutionary histories. Symbiotic genes show G-C content and codon usage patterns distinct from the rest of the genome in *Sinorhizobium meliloti* (Galibert et al., 2001), and homologous symbiotic genes occur in very distinct bacterial lineages, implying horizontal transfer, though from an as yet unknown source. The homologous system of dialog with legume hosts using Nod factors and of nitrogen fixation within nodules has been acquired by only ten distinct genera of alpha- and two distinct genera of beta-proteobacteria. Thus acquisition of nodulation induction, though repeated, appears rare at the level of bacterial genera (Remigi et al., 2016). Within genera, however, transfer of these genes appears to occur frequently. Symbiotic genes often show far less genetic diversity than do core genes, suggesting very recent horizontal transfer (Laranjo et al., 2008; Kumar et al., 2015), and congruent topologies of trees based on several different Nod genes (*nod*, *nol*, and *noe*) imply that these genes generally evolved together and are transferred among bacteria as a single unit (Moulin et al., 2004), although exceptions are known (Steenkamp et al., 2008). For example, two recently described species of *Bradyrhizobium* nodulating *Jicama*, *Pachyrhizus erosus*, in Central America closely resemble *Bradyrhizobium* strains isolated from soybean and *Vicia* for the *nifH* gene but are highly divergent from these strains for the *nodD* gene (Ramírez-Bahena et al., 2009), implying different evolutionary histories for these two types of symbiotic genes in these species.

**FIGURE 2:**
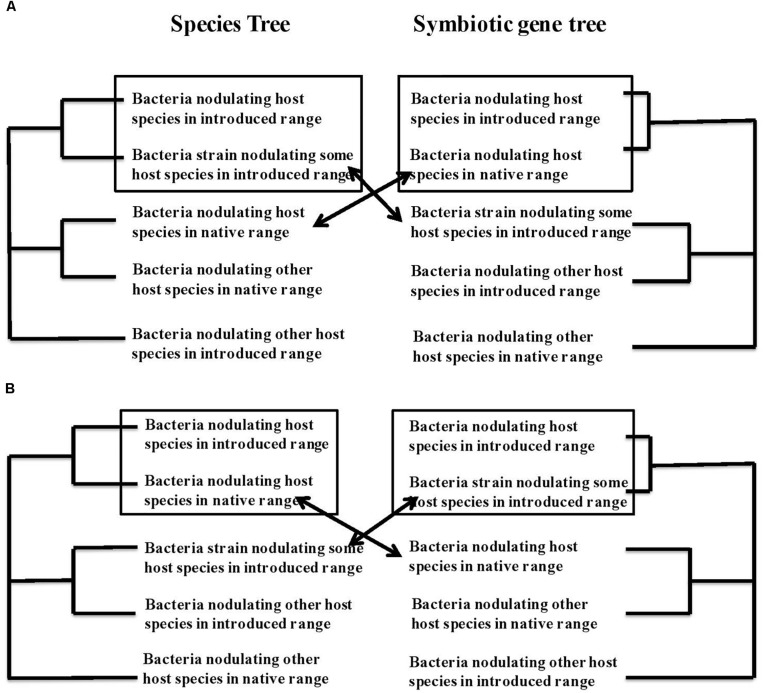
Phylogenetic trace of co-introduction or acquisition of a new symbiotic partner together with recombination between bacterial strains to generate chimeric symbiotic partners with core genetic markers and symbiotic genes that show different phylogenetic patterns. **(A)** Depicts what we subsequently call scenario 3, with a new symbiotic partner acquired in the new geographic range that resembles some local bacteria for core genes but that has recombined with the original symbiont to acquire the symbiotic genes specific to the original interaction, and thus resembles the original symbiotic partner at its symbiotic genes. **(B)** Depicts the opposite scenario, hereafter referred to as scenario 4 with co-introduced symbionts that resemble the bacteria from the native range of the host plant for core genes but has recombined to acquire new symbiotic genes from bacterial species present in the introduced range. As in Figure 1 we assume some geographic pattern to relationships of the bacteria, with species from the same geographic range showing more similarity.

Several events of apparently recent recombination have been observed between introduced and native microbes, such that the rhizobial bacteria are chimeric constructs, combining genetic features of co-introduced strains and strains derived from the bacterial communities at the site of introduction. Such recombination can be inferred from incongruence among the topologies of the gene genealogies of 16S and housekeeping genes on the one hand and symbiotic genes on the other for the same strains of bacteria. In Figure 2 we map out the possible types of recombination events and how they can be recognized from incongruences in the phylogenetic trees constructed from core or housekeeping genes versus symbiotic genes. Figure 2A shows the tree topologies consistent with the adoption of a new symbiont from the microbial community at the site of introduction that, however, bears the symbiotic machinery from the original bacterial partner. This is a pattern that makes ecological sense. If there is local adaptation in these soil microbes (Kraemer and Boynton, 2017) conditions in the new habitat may well be less favorable for bacteria from the original distribution than for local bacteria. If a local bacterium, adapted to the local conditions in the site of introduction, were to acquire the symbiotic genes involved in the dialog with the host plant and nitrogen fixation in the specific environment of its nodule, this new chimera would be adapted both to local abiotic conditions at the site and to the biotic interaction with this host species. Figure 2B shows another possible scenario of recombination.

There are several observations of introduced or invasive legumes nodulated by bacteria that are chimeras, bearing housekeeping genes of bacteria from the site of introduction but symbiotic genes of the habitual symbionts from the plants’ native ranges. Thus bacteria in the non-native ranges acquire the tools for interacting with introduced host plant from bacteria that must have been co-introduced. Indeed, genetic exchange requires that both types of bacteria, i.e., those from the native range of the host plant as well as those from the bacterial communities at the site of introduction co-occur at some point, hence presupposing co-introduction. For example, the invasive European *Cytisus scoparius* in the United States shows all three phenomena of co-introduction, acquisition of new symbiotic partners and chimeric bacterial symbionts. Some *Bradyrhizobium* species isolated from *C. scoparius* in the United States resemble those from European *C. scoparius*, some resemble strains that nodulate the native American *Lupinus lepidus*, indicating the acquisition of a native symbiont in the introduced range, and some are chimeric, with housekeeping genes characteristic of North American *Bradyrhizobium* but symbiotic genes identical to a single strain isolated from a *C. scoparius* growing in Spain (Horn et al., 2014). *R. pseudoacacia* in China is also nodulated by bacteria that resemble members of the Chinese rhizobial community at a number of genetic markers. These strains, however, bear *nodC* genes characteristic of strains that nodulate *R. pseudoacacia* in its native range (Wei et al., 2009), implying horizontal transfer of symbiotic genes from the habitual symbiont into the novel one adopted in the new distribution range. Other recombination patterns can also be observed, even in the absence of the habitual symbiotic partner, generating chimeric bacteria that combine features of two different bacteria both foreign to the introduced species. *Acacia mangium*, an Australian native has been planted throughout the tropics, where it can be nodulated by many local bacteria. In Brazil, at uninoculated sites this tree was nodulated by *Bradyrhizobium* that resemble *B. elkanii* strains from soybean, possibly from inoculum for their core genome. Their *nodA* gene sequences, however, resemble most closely bacteria that nodulate other *Acacia* species in Africa as well as other tropical legumes (Perrineau et al., 2011). Thus chimeric nodulating bacteria may be combinations of only novel foreign partners for introduced legume species.

Three of the most widely cultivated legumes are soybean, *Glycine max*, an Asian native, common bean, *Phaseolus vulgaris*, from Meso- and South America and peanut, *A. hypogaea*, originally from South America. Studies of the nodulating bacteria from soybean show both co-introduction and acquisition of novel bacterial strains from native plants in the introduced range. Soybean is usually inoculated at sowing, so finding apparently co-introduced rhizobia is unsurprising. For example, Shiro et al. (2013), studying nodulating strains from soybean throughout the United States, found that all of their isolated strains clustered together with reference strains from inocula, suggesting little implication of rhizobial strains from the North American soil bacterial community. Similarly, many effective nitrogen fixing strains isolated from the promiscuous soybean cultivar developed for cultivation in Africa to be compatible with African rhizobial bacteria were identified as *B. japonicum* or *B. elkanii* (Chibeba et al., 2017). On the other hand, some strains nodulating soybean in South Africa were distinct from known reference strains, suggesting that new strains had been adopted from the native soil community (Naamala et al., 2016). Other studies of soybean-nodulating bacteria from Brazil, Paraguay, and Canada recovered some strains nodulating soybean that resembled neither inoculum nor reference strains and that most likely had been acquired from native American *P. vulgaris* in South America (Chen et al., 2000; Hungria et al., 2001) or *Amphicarpaea bracteata* in Canada (Tang et al., 2012).

Common bean (*P. vulgaris*), a native of Central and South America, is now grown throughout the world as one of the most important legume crops. This promiscuous legume host can interact with a wide variety of bacterial species belonging mostly to the genus *Rhizobium* (Amarger, 2001). Within its centers of origin in Meso- and South America, beans are nodulated mainly by *Rhizobium etli* (Martínez-Romero, 2003). This bacterial species can also be found nodulating bean in introduced areas as widespread as Nepal (Adhikari et al., 2013), Ethiopia (Aserse et al., 2012), China (Cao et al., 2014), Tunisia (Mhamdi et al., 2002), Spain (Rodriguez-Navarro et al., 2000), Iran (Rouhrazi et al., 2016) and Jordan (Tamimi and Young, 2004). Throughout this plant’s range several other species of Rhizobia can be recovered from nodules. These include several closely related *Rhizobium* species, *R. tropici* (Martinez-Romero et al., 1991; Anyango et al., 1995; Grange and Hungria, 2004), *R. leguminosarum* (Adhikari et al., 2013; Ribeiro et al., 2013), but also bacteria belonging to the genera *Bradyrhizobium* (Han et al., 2005) and *Sinorhizobium* (Mnasri et al., 2007; Zurdo-Piñeiro et al., 2009). The overriding dominance of *R. etli* on the common bean throughout the world argues for a co-introduction scenario, unsurprising since bean seeds are known to carry *R. etli* (Pérez-Ramírez et al., 1998). On the other hand, the large spectrum of bacterial species that nodulate *Phaseolus* in its introduced ranges as well as their genetic distinctiveness indicates that this host species also regularly adopts new bacterial strains (Mwenda et al., 2018), sometimes from other crop species (Flores-Félix et al., 2018), in its area of introduction. In addition, bacterial strains nodulating *Phaseolus* can show incongruent phylogenetic relationships for housekeeping versus symbiotic genes, suggesting horizontal transfer of symbiotic genes (Aserse et al., 2012). For example, some rhizobial strains isolated from nodules of the common bean on Hispaniola Island resemble no other known bean nodulators for several housekeeping genes though they bear the typical *nodC* alleles of *R. phaseoli* (Díaz-Alcántara et al., 2014), suggesting adoption of new symbiotic partners that acquired the symbiotic genes adapted to interaction with this host.

The peanut (*A. hypogaea* L.) is the second most widely cultivated grain legume in the world, after soybean. This South America native, is nodulated by a large range of *Bradyrhizobium* and *Rhizobium* species in its native range (Taurian et al., 2006; Muñoz et al., 2011). Peanut is now an important oil and seed crop in several regions of Africa and Asia, where it is nodulated by several newly described species of bacteria (Wang et al., 2013; Grönemeyer et al., 2015, 2016; Li et al., 2015). One of these new species, *B. guangdongense*, clusters with *Bradyrhizobium* isolated from *Vigna unguiculata* from Brazil (Li et al., 2015), while the rest and additional strains from Ghana, resemble most closely *B. yuanmingense* originally isolated in China from *Lespedeza*, an Asian native, for housekeeping and 16S sequences (Wang et al., 2013; Grönemeyer et al., 2015, 2016; Li et al., 2015; Osei et al., 2018). This could suggest the acquisition of Asian bacteria after being introduced there, though how such strains arrived in Africa remains puzzling, as the strains isolated in Argentina, within the native range, also resemble these same species. Thus the source of bacteria that nodulate peanut in its introduced ranges in Asia and Africa remains unsolved and may point to a widespread cosmopolitan group of bacteria capable of effectively nodulating this promiscuous species. Isolates from Ghana appear to bear both a core genome and the symbiotic genes similar to those of *B. yuanmingense* from the host species *Lespedeza* (Osei et al., 2018) but *B. arachidis* from China has divergent and distinct *nifH* sequences (Wang et al., 2013), and both *B. guangxiense* and *B. guangdongense* have *nodA* genes identical to those reported from peanut isolates in Argentina (Muñoz et al., 2011; Li et al., 2015) suggesting recombination between housekeeping and symbiosis genes that generate chimeric strains as depicted in Figure 2A. On the other hand, strains isolated from peanut in South Africa and Botswana resemble most closely an inoculum strain widely deployed in Africa and originally isolated from the African native *Macrotyloma africanum* at a number of housekeeping genes. The various nod-genes appear to be of diverse African and Asian origin, suggesting that recombination has occurred, but that the nod-genes do not correspond to those from the native bacteria that had evolved in association with peanut (Steenkamp et al., 2008). Thus peanut nodulating bacteria also provide examples of unusual events that bring together novel, chimeric combinations of genes that had not previously evolved in the context of this host species, as depicted in Figure 2B. To further explore the interesting case of peanut nodulators, we constructed phylogenetic trees of 16S and housekeeping genes and compared these with trees constructed with the *nodC* and *nifH* gene sequences for those strains of peanut nodulators for which all sequence data were available. This allows us to explore the various scenarios found for these bacteria.

## Materials and Methods

We searched the National Center for Biotechnology Information (NCBI) database for the following keywords: *Bradyrhizobium* AND (peanut or *Arachis* or groundnut) to find sequence data of *Bradyrhizobium* associated with peanut (*A. hypogaea*). We retained only those 67 strains of *Bradyrhizobium* nodulating peanut for which we had the maximum of sequence data, in this case 16S ribosomal DNA, three housekeeping genes (*recA, dnaK, glnII*) and the symbiotic genes *nodC* and *nifH*. Almost all of these *Bradyrhizobium* strains were from China, with one from India. Sequence data were saved as a FASTA file. We added sequences data of the same genomic regions or genes provided by Mariangela Hungria from 28 reference strains that are commonly used in phylogenetic studies of *Bradyrhizobium*, for example, the recent publication of Delamuta et al. (2017). Two of these reference strains had been isolated from peanut, one from Namibia and one from China. Information on all reference strains and peanut associated strains are presented in Supplementary Table S1.

FASTA files were aligned with the reference strains mentioned above using T-COFFEE, retaining the original reference alignment (-profile method, Notredame et al., 2000). Aligned and trimmed sequences of 16S rRNA and housekeeping genes (*recA, dnaK, glnII*) were concatenated in bash.

Maximum likelihood trees were constructed based on each of the sequences independently and on the concatenated sequence alignment of 16S plus housekeeping genes using 100 bootstrap replicated in RAxML 8.2.7 (-f a -# 100, Stamatakis, 2014). Branch support was assessed using the transfer bootstrap expectation (Lemoine et al., 2018) as implemented by Booster^1^. Branch lengths, presented only in the Supplementary Figures, were calculated by Maximum Likelihood under a GTR-Gamma model in RAxML. Congruence among trees was tested with an approximate unbiased (AU) test, implemented in CONSEL (Shimodaira, 2002).

## Results

**FIGURE 3:**
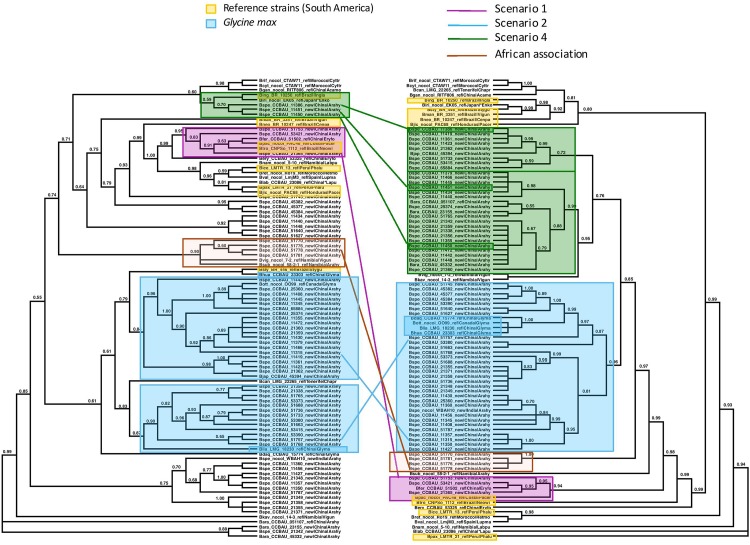
Maximum Likelihood trees of the concatenated sequences of 16S region and the housekeeping genes r*ecA, dnaK, glnII* on the left, labeled “Species Tree”, and on the right, of the symbiotic *nifH* gene. Nodes with lower bootstrap support than 60% were collapsed into polytomies. Bootstrap support is coded for each node. The two trees differ significantly in topology (Approximate Unbiased (AU) test *p* < 0.05). We identified clades or clusters of strains of bacteria on the two trees that illustrated the different scenarios outlined in Figures 1, 2. Clades or clusters are identified with a color-coding by their host plant, and different colored lines to illustrate the different scenarios join individual strains or clades on the two trees. When more than one member of a clade or cluster followed the same evolutionary scenario we chose a single one to illustrate it. The scenarios shown do not represent all events that can be traced from this phylogeny, but are representative of the events that it elucidates. We also mark a clade of bacteria isolated from peanut in China that cluster with Namibian bacterial isolates on the species tree but form an isolated clade that includes no reference strains on the *nifH* tree.

**FIGURE 4:**
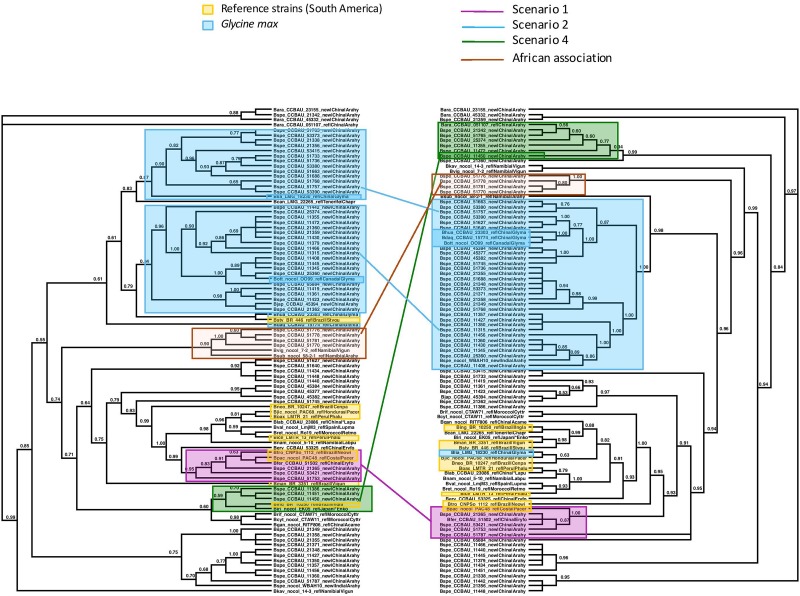
Maximum Likelihood trees of the concatenated sequences of 16S region and the housekeeping genes r*ecA, dnaK, glnII* on the left, labeled “Species Tree”, and on the right, of the symbiotic *nodC* gene. Nodes with lower bootstrap support than 60% were collapsed into polytomies. Bootstrap support is coded for each node. The two trees differ significantly in topology (AU test *p* < 0.05). We identified clades or clusters of strains of bacteria on the two trees that illustrated the different scenarios outlined in Figures 1, 2. Clades or clusters are identified with a color-coding by their host plant, and different colored lines to illustrate the different scenarios join individual strains or clades on the two trees. When more than one member of a clade or cluster followed the same evolutionary scenario we chose a single one to illustrate it. The scenarios shown do not represent all events that can be traced from this phylogeny, but are representative of the events that it elucidates. We also mark a clade of bacteria isolated from peanut in China that cluster with Namibian bacterial isolates on the species tree but form an isolated clade that includes no reference strains on the *nodC* tree.

Although the trees based on individual sequences of 16S or housekeeping genes were poorly resolved, with more than 70% of the internal branches collapsing when applying a 75% threshold, the ML tree based on the concatenation of the 16S and housekeeping genes was better resolved (fewer than 30% of the branches collapsed at the same threshold). For the *nifH* tree, 41% of the branches collapsed when applying a 75% threshold, but these were almost always terminal branches, as is obvious from the terminal polytomies in the *nifH* tree in Figure 3. For the *nodC* tree, almost 30% of the branches collapsed when applying a 75% threshold, but these were likewise almost always terminal branches, as is obvious from the terminal polytomies in the *nodC* tree in Figure 4.

Reciprocal AU tests revealed that only a minority (fewer than 40%) of the bootstrap replicates of 16S and housekeeping gene trees were significantly (AU *p* < 0.05) incongruent with one-another or with the concatenation tree. The two symbiotic gene trees based on the full sequence data set were not fully congruent, showing some evidence of recombination between *nodC* and *nifH* genes (Figure 4). However, after collapsing branches with transfer bootstrap expectation smaller than 50% *the nifH* and *nodC* gene trees were not significantly incongruent (smallest AU *p* = 0.08). All of the ML trees and bootstrap replicates showed significant incongruence between the concatenation tree and the *nifH* tree (AU *p* < 0.05 for all comparisons). Because the *nodC* and the nifH collapsed trees were not incongruent, this implies that the *nodC* tree was also incongruent with the concatenated tree. Therefore, the symbiotic genes shows an evolutionary history that is distinct from that of the other genes, which, together, tell a congruent evolutionary story. Figure 3 compares the topologies of the trees based on the concatenation of the 16S and housekeeping genes with the *nifH* tree. Figure 4 compares the topologies of the trees based on the concatenation of the 16S and housekeeping genes with the *nodC* tree. Below we examine more closely several of the incongruences among the concatenated and the symbiotic gene trees. Tree comparisons including branch lengths that reflect substitutions per site are presented in Supplementary Figure S1 (concatenated versus *nifH*), Supplementary Figure S2 (concatenated versus *nodC*), and Supplementary Figure S3 (*nifH* versus *nodC*).

We examined the trees of the peanut-nodulating strains to find cases of the different scenarios sketched in Figures 1, 2. In Figures 3, 4 clades or clusters of bacterial strains that can be attributed to a particular host species on either tree are color-coded. Strains that illustrate one of the evolutionary scenarios we described in Figures 1, 2 are marked. When several strains in any clade illustrate the same scenario we mark only one as an example. Thus Figures 3, 4 summarize our interpretation of the relationships among the different strains and how this elucidates the history of the association between the introduced peanut and the rhizobial strains it associates with.

We did not have the complete set of all gene sequences for any strains isolated from peanut in the native South America available to include in the analyses. However, there are several reference strains of bacteria from native South American species. When peanut rhizobia cluster together with these on either the concatenated or the symbiotic gene trees we consider this evidence that the strains were introduced from the native area of peanut, having a South American origin, thereby representing the scenario 1. Some strains isolated from peanut in China resembled most closely a strain from the native South American legume *Pachyrrhizus* on both the concatenated gene tree and the symbiotic gene trees, thus we consider this a case of co-introduction of peanut with a South American bacterial symbiont, marked in pink on Figures 3, 4. The large majority of strains isolated from peanut in China resembled most closely bacterial strain nodulating soybean in China for both housekeeping and symbiotic genes, thus presenting cases of scenario 2, the adoption of a new rhizobial partner in the introduced area, marked in blue on Figures 3, 4.

We found no cases consistent with scenario 3 (Figure 2A) but, surprisingly we found a case of scenario 4, depicted in Figure 2B, of what appears to be a co-introduction of peanut with a bacterial strain that nodulates *Inga* in Brazil that recombined with bacteria similar to those nodulating soybean, leading to a recombinant with the symbiotic genes most closely related to those of soybean strains combined with the housekeeping genes of a strain from Brazil (marked in green on Figures 3, 4).

Some clades of bacterial strains isolated from peanut in China cluster together with no reference strains. Hence their origin remains unclear. However, it appears that a majority of peanut rhizobial strains in China are similar to strains that nodulate soybean. For example, some peanut strains cluster with two reference strains of new species described from Namibia, one from peanut and one from Bambara groundnut. These reference strains, however, were described as very similar to strains from soybean for both housekeeping and symbiotic genes (Grönemeyer et al., 2015).

## Discussion

What is the nature of the bacterial symbionts of the legumes that human agricultural practices have moved around the world? In the literature we found most examples of plant species that appeared to have been co-introduced with their nodulating bacteria, i.e., for which the strains isolated in the introduced range were most similar to strains from the original native range. This is unsurprising for legume species that are inoculated either systematically or when they are first planted in a new area (Stȩpkowski et al., 2005) and soybean is systematically inoculated in large parts of its cultivation area (Chang et al., 2015). The importance of soil bacteria as plant growth promotors and in nitrogen fixation in the root nodules of many legumes has been known since the late 19th century and inoculation was practiced even before people understood why (Brown, 1918). This long history of moving soil together with plants could well explain why many introduced legumes can be nodulated by their habitual bacteria from their native range. Similarly, unintentional introduction or invasion that was initiated from soil contaminated with seed of a non-native species would have been likely to also include their nodulating bacteria. A co-introduction scenario is thus compatible with the many observations of introduced plants associating with bacteria that strongly resemble their native symbionts, even in the introduced range. On the other hand, the close similarity between bacteria that nodulate an introduced species in its native and introduced range could, alternatively, indicate that bacteria very similar to their habitual nodulators were already present at the site of introduction. Indeed, distinguishing between a scenario of co-introduction and the “Everything is everywhere but…” hypothesis of Baas-Becking (de Wit and Bouvier, 2006) is virtually impossible.

It was a far greater challenge to find examples of plants that were NOT co-introduced with their symbionts, or for which the symbiont in the introduced range resembled more closely the symbionts of other native species in that range. The single case we found, of *M. pigra* in Australia (Parker et al., 2007), does not even completely meet our criteria, because we have no phylogenetic information for the *Burkholderia* that associate with other species in the introduced range. Nonetheless. it is clear that the lineages of this bacteria that associate with *M. pigra* in Australia are distinct from those in the neotropics, the native range of these plants. Co-introduction does not preclude additional associations with members of the new bacterial community. We found numerous examples of introduced plant species that associate both with their habitual symbionts but that have clearly adopted additional bacterial strains that are distinct from those found in their native range and that resemble bacteria from the soil community of the introduced range (Chen et al., 2000; Ulrich and Zaspel, 2000; Hungria et al., 2001; Wei et al., 2009; Tang et al., 2012; Ndlovu et al., 2013; Chibeba et al., 2017).

The more surprising result, perhaps, was the amount of recombination and lateral gene transfer evident in the bacterial symbionts of these plants. Such lateral gene transfers and exchanges of symbiotic genes is already discussed in the literature (Steenkamp et al., 2008; Ramírez-Bahena et al., 2009; Wei et al., 2009; Muñoz et al., 2011; Perrineau et al., 2011; Aserse et al., 2012; Díaz-Alcántara et al., 2014; Horn et al., 2014). Our ability to even ask these questions about the frequency and nature of chimeric bacterial strains has been made possible by technological breakthroughs that allow rapid sequencing at low cost that provide sequence data on multiple genetic markers per individual. Indeed, few publications on bacterial diversity from before the current millenium presented data from multiple markers, which made it impossible to even address these questions.

Based on the strains of *Bradyrhizobium* isolated from peanut for which we had adequate sequence data for our analyses, we found poor congruence between the tree based on 16S and housekeeping genes and both the *nodC* and *nifH* trees. Though such incongruence is not always found (Jaiswal et al., 2017) it is a general finding for these and other strains (Menna and Hungria, 2011; Zinga et al., 2017; Tong et al., 2018) implying widespread recombination with horizontal exchange of the symbiotic mechanism, as has been noted on both large and small scales in these bacteria (Galibert et al., 2001; Kumar et al., 2015; Remigi et al., 2016). Indeed, from our analysis of these peanut-nodulating strains mostly from China, we found patterns that were consistent with co-introduction of bacteria from the native range, with adoption of new strains and novel associations in the introduced range and with recombination that generated new, chimeric symbionts.

Surprisingly, we found no cases of recombination with adopted novel symbionts acquiring the symbiotic mechanisms from the habitual nodulating strains, which we had expected to be the usual case for chimeric bacteria. Indeed one would expect chimeric strains that combine the genes responsible for dialog with the host plant that are conserved from the original association, but with genes responsible for adaptation to the abiotic environment to be acquired from the local bacterial community at the site of introduction. Such a pattern has been found for bacteria associated with several plant species including peanut, with bacteria resembling species from the local rhizobial community in the introduced range for housekeeping genes but resembling the original nodulating bacteria from the plants’ native range for their symbiotic genes (Wei et al., 2009; Díaz-Alcántara et al., 2014; Horn et al., 2014; Li et al., 2015; Wang et al., 2016). Here, on the other hand, *nodC* and *nifH* genes similar to those from the native range in South America were not found in other genetic backgrounds of bacteria adopted from the local communities. However, there were cases of peanut-nodulating bacteria that clustered together with bacterial strains from their native South America for housekeeping genes but, for their symbiotic genes, were most similar to strains that have symbiotic genes like those of bacteria nodulating soybean. For example, *B. japonicum* was isolated from soybean and *B. arachidis*, a bacteria associated with peanut first described by Wang et al. (2013), has *nodC* and *nifH* genes that cluster with strains from soybean. Thus we identified bacterial symbionts of peanut in China that combined the housekeeping genes of an introduced bacterium foreign to Chinese soils with symbiotic genes new for peanut.

One group of peanut-nodulating bacteria isolated in China formed a clade, on the species tree, with *B. subterraneum* isolated from peanut and *B. vignae*, isolated from *V. unguiculata*, both from Namibia, marked as “African association” on Figures 3, 4. For the *nodC* and *nifH* genes the group of peanut isolates formed an isolated clade on its own. The close relationship with bacterial isolates from Namibia poses an interesting problem. Either these strains colonized China via southern Africa, or vice versa. Peanut cultivation in China may be very old. Though peanut originated in the new world, and as such should have been unknown outside of the Americas before the Columbian Exchange, it is unambiguously mentioned in Chinese horticultural literature in the early sixteenth century, which seems too soon to have followed a European route of introduction to China. This suggests that peanuts may have arrived in China in pre-Columbian times, possibly via a western route out of South America and Chinese mariners’ voyages of discovery of the islands of the South China Sea (Ho, 1955). Thus it is not impossible that peanut was first cultivated in China and subsequently moved to Africa, possibly bringing with it the bacteria it had adopted in China.

This analysis and interpretation of the available genomes of peanut-associated rhizobia, though a valid first step, has failed in clearly elucidating the origin of the bacterial symbionts of peanut in China, let alone in other introduced areas. Furthermore, though we were able to identify events of host shift and recombination, our approach was mainly qualitative, i.e., particular events could be observed or not. Understand the evolutionary forces and processes that shape this complex interaction between host and symbiont would necessitate a more quantitative approach to determine how often particular phenomena occur. The latter requires a population genetics approach, which should become increasingly accessible through technological advances.

We are left with a large number of intriguing questions about peanut-nodulators, which of course can be posed for any species of legume that has spread beyond its native range: What is the origin of the symbiotic bacteria, and for which genes, the core genome or the symbiotic genes? Can bacterial phylogeography elucidate patterns of agricultural exchange? How do abiotic conditions influence host range for bacteria and symbiont choice for the plants? How broad is the host range of bacterial strains that can nodulate peanut. How interchangeable are symbiotic genes? How large is the range of core genomes in which symbiotic genes can function? Does the compatibility between core and symbiotic genes influence symbiotic efficiency? Is the unexpected extent of the recombination and the acquisition of nitrogen-fixation genes from divers sources an exception? Is the case of peanuts unusual or are many symbiotic bacteria comprised of chimeric populations of such diverse origins for symbiotic as well as core genes?

Addressing these questions would require far more extensive sampling of bacteria that nodulate peanut as well as a large range of native species that could harbor the sources of peanut-nodulating bacteria in the introduced ranges. Additional sampling of bacterial symbionts of a range of host plants as well as of peanuts from more introduced areas would improve the scope of the study and allow us to explore the intriguing patterns we uncovered of rampant recombination in all directions in the bacteria nodulating this promiscuous host species in its introduced range. Peanut rhizobia, at least in China, appears to present a convoluted history of host shift and recombination to an unusual degree. Only more systematic study of peanut, but also other bacterial symbionts of widespread crop and introduced species confronted with bacterial diversity with which they have not evolved, can answer whether this is a rare or a habitual occurrence. Though the complete sampling of adequate representatives of all potential sources of bacteria is probably out of reach, we hope however, to stimulate a more systematic approach to this question.

## Author Contributions

BB, IG, BA, and JS thought up and wrote the manuscript. RR carried out the bioinformatics analyses, and discussed the results and their interpretation. MH provided the sequence data. All authors reviewed and refined the manuscript.

## Conflict of Interest Statement

The authors declare that the research was conducted in the absence of any commercial or financial relationships that could be construed as a potential conflict of interest.

## Footnotes

1 https://github.com/evolbioinfo/booster

## Supplementary Material

FIGURE S1 Presents the Maximum Likelihood trees, with branch lengths measured in substitutions per site, calculated under a GTR-Gamma model in RAxML, of the concatenated sequences of 16S region and the housekeeping genes *recA, dnaK, glnII* on the left, labeled “Species Tree”, and on the right, of the symbiotic *nifH* gene.

FIGURE S2 Presents the Maximum Likelihood trees, with branch lengths measured in substitutions per site, calculated under a GTR-Gamma model in RAxML, of the concatenated sequences of 16S region and the housekeeping genes r*ecA, dnaK, glnII* on the left, labeled “Species Tree”, and on the right, of the symbiotic *nodC* gene.

FIGURE S3 Presents the Maximum Likelihood trees, with branch lengths measured in substitutions per site, calculated under a GTR-Gamma model in RAxML, of *nifH* on the left and *nodC* on the right.

TABLE S1 Presents a list of all strains used.

## References

[B1] Adhikari D. Itoh K. Suyama K. (2013). Genetic diversity of common bean (*Phaseolus vulgaris* L.) nodulating rhizobia in Nepal. *Plant Soil* 368 341–353. 10.1007/s11104-012-1518-7

[B2] Amarger N. (2001). Rhizobia in the field. *Adv. Agron.* 73 109–168. 10.1016/s0065-2113(01)73006-4

[B3] Anyango B. Wilson K. J. Beynon J. L. Giller K. E. (1995). Diversity of rhizobia nodulating *Phaseolus vulgaris* L. in two Kenyan soils with contrasting pHs. *Appl. Environ. Microbiol.* 61 4016–4021. 16535165 10.1128/aem.61.11.4016-4021.1995 PMC1388601

[B4] Aserse A. A. Räsänen L. A. Assefa F. Hailemariam A. Lindström K. (2012). Phylogeny and genetic diversity of native rhizobia nodulating common bean (*Phaseolus vulgaris* L.) in Ethiopia. *Syst. Appl. Microbiol.* 35 120–131. 10.1016/j.syapm.2011.11.005 22265597

[B5] Brown P. E. (1918). Soil inoculation. *Circular* 43 1–7. 10.1016/j.expneurol.2008.02.004 18423451

[B6] Cao Y. Wang E. T. Zhao L. Chen W. M. Wei G. H. (2014). Diversity and distribution of rhizobia nodulated with *Phaseolus vulgaris* in two ecoregions of China. *Soil Biol. Biochem.* 78 128–137. 10.1016/j.soilbio.2014.07.026

[B7] Chang W.-S. Lee H.-I. Hungria M. (2015). “Soybean production in the americas,” in *Principles of Plant-Microbe Interactions: Microbes for Sustainable Agriculture*, ed. Lugtenberg B. (Cham: Springer), 393–400. 10.1007/978-3-319-08575-3_41

[B8] Chen L. S. Figueredo A. Pedrosa F. O. Hungria M. (2000). Genetic characterization of soybean rhizobia in Paraguay. *Appl. Environ. Microbiol.* 66 5099–5103. 10.1128/AEM.66.11.5099-5103.2000 11055970 PMC92426

[B9] Chen W. M. James E. K. Chou J. H. Sheu S. Y. Yang S. Z. Sprent J. I. (2005). β-rhizobia from *Mimosa pigra*, a newly discovered invasive plant in Taiwan. *New Phytol.* 168 661–675. 10.1111/j.1469-8137.2005.01533.x 16313648

[B10] Chibeba A. M. Kyei-Boahen S. Guimarães M. de F. Nogueira M. A. Hungria M. (2017). Isolation, characterization and selection of indigenous *Bradyrhizobium* strains with outstanding symbiotic performance to increase soybean yields in Mozambique. *Agric. Ecosyst. Environ.* 246 291–305. 10.1016/j.agee.2017.06.017 28775390 PMC5521954

[B11] de Wit R. Bouvier T. (2006). ‘Everything is everywhere, but, the environment selects’; what did baas becking and beijerinck really say? *Environ. Microbiol.* 8 755–758. 10.1111/j.1462-2920.2006.01017.x 16584487

[B12] Delamuta J. R. M. Menna P. Ribeiro R. A. Hungria M. (2017). Phylogenies of symbiotic genes of *Bradyrhizobium* symbionts of legumes of economic and environmental importance in Brazil support the definition of the new symbiovars *pachyrhizi* and sojae. *Syst. Appl. Microbiol.* 40 254–265. 10.1016/j.syapm.2017.04.005 28647304

[B13] Díaz-Alcántara C. A. Ramírez-Bahena M. H. Mulas D. García-Fraile P. Gómez-Moriano A. Peix A. (2014). Analysis of rhizobial strains nodulating *Phaseolus vulgaris* from Hispaniola island, a geographic bridge between meso and South America and the first historical link with Europe. *Syst. Appl. Microbiol.* 37 149–156. 10.1016/j.syapm.2013.09.005 24239274

[B14] Flores-Félix J. D. Sánchez-Juanes F. García-Fraile P. Valverde A. Mateos P. F. Gónzalez-Buitrago J. M. (2018). *Phaseolus vulgaris* is nodulated by the symbiovar viciae of several genospecies of rhizobium laguerreae complex in a Spanish region where Lens culinaris is the traditionally cultivated legume. *Syst. Appl. Microbiol.* 42 240–247. 10.1016/j.syapm.2018.10.009 30415881

[B15] Galibert F. Finan T. M. Long S. R. Pühler A. Abola P. Ampe F. (2001). The composite genome of the legume symbiont *Sinorhizobium meliloti*. *Science* 293 668–672. 10.1126/science.1060966 11474104

[B16] Grange L. Hungria M. (2004). Genetic diversity of indigenous common bean (*Phaseolus vulgaris*) rhizobia in two Brazilian ecosystems. *Soil Biol. Biochem.* 36 1389–1398. 10.1016/j.soilbio.2004.03.005

[B17] Grönemeyer J. L. Chimwamurombe P. Reinhold-Hurek B. (2015). *Bradyrhizobium subterraneum* sp. nov., a symbiotic nitrogen-fixing bacterium from root nodules of groundnuts. *Int. J. Syst. Evol. Microbiol.* 65 3241–3247. 10.1099/ijsem.0.000403 26198108

[B18] Grönemeyer J. L. Hurek T. Bünger W. Reinhold-Hurek B. (2016). *Bradyrhizobium vignae* sp. nov., a nitrogen-fixing symbiont isolated from effective nodules of *Vigna* and *Arachis*. *Int. J. Syst. Evol. Microbiol.* 66 62–69. 10.1099/ijsem.0.000674 26463703

[B19] Han S. Z. Wang E. T. Chen W. X. (2005). Diverse bacteria isolated from root nodules of *Phaseolus vulgaris* and species within the genera *Campylotropis* and *cassia* grown in China. *Syst. Appl. Microbiol.* 28 265–276. 10.1016/j.syapm.2004.12.005 15900972

[B20] Ho P.-T. (1955). The introduction of american food plants into China. *Am. Anthropol.* 57 191–201. 10.1016/j.amepre.2015.06.010 26321012 PMC4691550

[B21] Horn K. Parker I. M. Malek W. Rodríguez-Echeverría S. Parker M. A. (2014). Disparate origins of *Bradyrhizobium* symbionts for invasive populations of *Cytisus scoparius* (Leguminosae) in North America. *FEMS Microbiol. Ecol.* 89 89–98. 10.1111/1574-6941.12335 24712882

[B22] Hungria M. Lígia L. M. Coca R. G. Megías M. (2001). Preliminary characterization of fast growing rhizobial strains isolated from soyabean nodules in Brazil. *Soil Biol. Biochem.* 33 1349–1361. 10.1016/S0038-0717(01)00040-2

[B23] Jaiswal S. K. Msimbira L. A. Dakora F. D. (2017). Phylogenetically diverse group of native bacterial symbionts isolated from root nodules of groundnut (*Arachis hypogaea* L.) in South Africa. *Syst. Appl. Microbiol.* 40 215–226. 10.1016/j.syapm.2017.02.002 28372899 PMC5460907

[B24] Karimi B. Terrat S. Dequiedt S. Saby N. P. A. Horrigue W. Lelièvre M. (2018). Biogeography of soil bacteria and archaea across France. *Sci. Adv.* 4:eaat1808. 10.1126/sciadv.aat1808 29978046 PMC6031370

[B25] Kraemer S. A. Boynton P. J. (2017). Evidence for microbial local adaptation in nature. *Mol. Ecol.* 26 1860–1876. 10.1111/mec.13958 27997057

[B26] Kumar N. Lad G. Giuntini E. Kaye M. E. Udomwong P. Jannah Shamsani N. (2015). Bacterial genospecies that are not ecologically coherent: population genomics of *Rhizobium leguminosarum*. *Open Biol.* 5:140133. 10.1098/rsob.140133 25589577 PMC4313370

[B27] Laranjo M. Alexandre A. Rivas R. Velázquez E. Young J. P. W. Oliveira S. (2008). Chickpea rhizobia symbiosis genes are highly conserved across multiple *Mesorhizobium* species. *FEMS Microbiol. Ecol.* 66 391–400. 10.1111/j.1574-6941.2008.00584.x 18795953

[B28] Lemoine F. Domelevo Entfellner J.-B. Wilkinson E. Correia D. Davila Felipe M. De Oliveira T. (2018). Renewing felsenstein’s phylogenetic bootstrap in the era of big data. *Nature* 556 452–456. 10.1038/s41586-018-0043-0 29670290 PMC6030568

[B29] Li Y. H. Wang R. Zhang X. X. Young J. P. W. Wang E. T. Sui X. H. (2015). *Bradyrhizobium guangdongense* sp. nov. and *Bradyrhizobium guangxiense* sp. nov., isolated from effective nodules of peanut. *Int. J. Syst. Evol. Microbiol.* 65 4655–4661. 10.1099/ijsem.0.000629 26409482

[B30] Martínez-Romero E. (2003). Diversity of *Rhizobium*-*Phaseolus vulgaris* symbiosis: overview and perspectives. *Plant Soil* 252 11–23. 10.1023/A:1024199013926

[B31] Martinez-Romero E. Segovia L. Mercante F. M. Franco A. A. Graham P. Marco A. (1991). *Rhizobium tropici*, a novel species nodulating *Phaseolus vulgaris* L. beans and Leucaena sp. trees. *Int. J. Syst. Bacteriol.* 41 417–426. 10.1099/00207713-41-3-417 1715738

[B32] Menna P. Hungria M. (2011). Phylogeny of nodulation and nitrogen-fixation genes in *Bradyrhizobium*: supporting evidence for the theory of monophyletic origin, and spread and maintenance by both horizontal and vertical transfer. *Int. J. Syst. Evol. Microbiol.* 61 170–172. 10.1099/ijs.0.028803-0 21357454

[B33] Mhamdi R. Laguerre G. Aouani M. E. Mars M. Amarger N. (2002). Different species and symbiotic genotypes of field rhizobia can nodulate *Phaseolus vulgaris* in tunisian soils. *FEMS Microbiol. Ecol.* 41 77–84. 10.1111/j.1574-6941.2002.tb00968.x 19709241

[B34] Mnasri B. Mrabet M. Laguerre G. Aouani M. E. Mhamdi R. (2007). Salt-tolerant rhizobia isolated from a Tunisian oasis that are highly effective for symbiotic N2-fixation with Phaseolus vulgaris constitute a novel biovar (bv. mediterranense) of *Sinorhizobium meliloti*. *Arch. Microbiol.* 187 79–85. 10.1007/s00203-006-0173-x 17019605

[B35] Moulin L. Béna G. Boivin-Masson C. Stepkowski T. (2004). Phylogenetic analyses of symbiotic nodulation genes support vertical and lateral gene co-transfer within the *Bradyrhizobium* genus. *Mol. Phylogenet. Evol.* 30 720–732. 10.1016/S1055-7903(03)00255-0 15012950

[B36] Muñoz V. Ibañez F. Tonelli M. L. Valetti L. Anzuay M. S. Fabra A. (2011). Phenotypic and phylogenetic characterization of native peanut *Bradyrhizobium* isolates obtained from Córdoba. *Argentina. Syst. Appl. Microbiol.* 34 446–452. 10.1016/j.syapm.2011.04.007 21742454

[B37] Mwenda G. M. O’Hara G. W. De Meyer S. E. Howieson J. G. Terpolilli J. J. (2018). Genetic diversity and symbiotic effectiveness of *Phaseolus vulgaris*-nodulating rhizobia in Kenya. *Syst. Appl. Microbiol.* 41 291–299. 10.1016/j.syapm.2018.02.001 29571921 PMC6052332

[B38] Naamala J. Jaiswal S. K. Dakora F. D. (2016). Microsymbiont diversity and phylogeny of native *Bradyrhizobia* associated with soybean (*Glycine max* L. Merr.) nodulation in South African soils. *Syst. Appl. Microbiol.* 39 336–344. 10.1016/j.syapm.2016.05.009 27324571 PMC4958686

[B39] Ndlovu J. Richardson D. M. Wilson J. R. U. Le Roux J. J. (2013). Co-invasion of South African ecosystems by an australian legume and its rhizobial symbionts. *J. Biogeogr.* 40 1240–1251. 10.1111/jbi.12091

[B40] Notredame C. Higgins D. G. Heringa J. (2000). T-coffee: a novel method for fast and accurate multiple sequence alignment. *J. Mol. Biol.* 302 205–217. 10.1006/jmbi.2000.4042 10964570

[B41] Osei O. Abaidoo R. C. Ahiabor B. D. K. Boddey R. M. Rouws L. F. M. (2018). Bacteria related to *Bradyrhizobium yuanmingense* from Ghana are effective groundnut micro-symbionts. *Appl. Soil Ecol.* 127 41–50. 10.1016/j.apsoil.2018.03.003 PMC5989812 29887673

[B42] Parker M. A. (2001). Mutualism as a constraint on invasion success for legumes and rhizobia. *Divers. Distrib.* 7 125–136. 10.1046/j.1472-4642.2001.00103.x

[B43] Parker M. A. Wurtz A. K. Paynter Q. (2007). Nodule symbiosis of invasive *Mimosa pigra* in Australia and in ancestral habitats: a comparative analysis. *Biol. Invasions* 9 127–138. 10.1007/s10530-006-0009-2

[B44] Pérez-Ramírez N. O. Rogel M. A. Wang E. Castellanos J. Z. Martínez-Romero E. (1998). Seeds of *Phaseolus vulgaris* bean carry *Rhizobium etli*. *FEMS Microbiol. Ecol.* 26 289–296. 10.1139/w10-048 20725128

[B45] Perrineau M. M. Le Roux C. de Faria S. M. de Carvalho Balieiro F. Galiana A. Prin Y. (2011). Genetic diversity of symbiotic *Bradyrhizobium elkanii* populations recovered from inoculated and non-inoculated *Acacia mangium* field trials in Brazil. *Syst. Appl. Microbiol.* 34 376–384. 10.1016/j.syapm.2011.03.003 21531520

[B46] Ramírez-Bahena M. H. Peix A. Rivas R. Camacho M. Rodríguez-Navarro D. N. Mateos P. F. (2009). *Bradyrhizobium pachyrhizi* sp. nov. and *Bradyrhizobium jicamae* sp. nov., isolated from effective nodules of *Pachyrhizus erosus*. *Int. J. Syst. Evol. Microbiol.* 59 1929–1934. 10.1099/ijs.0.006320-0 19567584

[B47] Remigi P. Zhu J. Young J. P. W. Masson-Boivin C. (2016). Symbiosis within symbiosis: evolving nitrogen-fixing legume symbionts. *Trends Microbiol.* 24 63–75. 10.1016/j.tim.2015.10.007 26612499

[B48] Ribeiro R. A. Ormeño-Orrillo E. Dall’Agnol R. F. Graham P. H. Martinez-Romero E. Hungria M. (2013). Novel *Rhizobium* lineages isolated from root nodules of the common bean (*Phaseolus vulgaris* L.) in Andean and Mesoamerican areas. *Res. Microbiol.* 164 740–748. 10.1016/j.resmic.2013.05.002 23764913

[B49] Rodríguez-Echeverría S. Fajado S. Ruiz-Díez B. Fernández-Pascual M. (2012). Differential effectiveness of novel and old legume-rhizobia mutualisms: implications for invasion by exotic legumes. *Oecologia* 170 253–261. 10.1007/s00442-012-2299-7 22419481

[B50] Rodriguez-Navarro D. N. Buendia A. M. Camacho M. Lucas M. M. Santamaria C. (2000). Characterization of *Rhizobium* spp. bean isolates from South-West Spain. *Soil Biol. Biochem.* 32 1601–1613. 10.1016/S0038-0717(00)00074-2

[B51] Rouhrazi K. Khodakaramian G. Velázquez E. (2016). Phylogenetic diversity of rhizobial species and symbiovars nodulating *Phaseolus vulgaris* in Iran. *FEMS Microbiol. Lett.* 363 1–8. 10.1093/femsle/fnw024 26832644

[B52] Shimodaira H. (2002). An approximately unbiased test of phylogenetic tree selection. *Syst. Biol.* 51 492–508. 10.1080/10635150290069913 12079646

[B53] Shiro S. Matsuura S. Saiki R. Sigua G. C. Yamamoto A. Umehara Y. (2013). Genetic diversity and geographical distribution of indigenous soybean-nodulating *Bradyrhizobia* in the United States. *Appl. Environ. Microbiol.* 79 3610–3618. 10.1128/AEM.00236-3 23563944 PMC3675916

[B54] Simonsen A. K. Dinnage R. Barrett L. G. Prober S. M. Thrall P. H. (2017). Symbiosis limits establishment of legumes outside their native range at a global scale. *Nat. Commun.* 8 1–9. 10.1038/ncomms14790 28387250 PMC5385628

[B55] Stamatakis A. (2014). RAxML version 8: a tool for phylogenetic analysis and post-analysis of large phylogenies. *Bioinformatics* 30 1312–1313. 10.1093/bioinformatics/btu033 24451623 PMC3998144

[B56] Steenkamp E. T. Stepkowski T. Przymusiak A. Botha W. J. Law I. J. (2008). Cowpea and peanut in southern Africa are nodulated by diverse *Bradyrhizobium* strains harboring nodulation genes that belong to the large pantropical clade common in Africa. *Mol. Phylogenet. Evol.* 48 1131–1144. 10.1016/j.ympev.2008.04.032 18539053

[B57] Stepkowski T. Moulin L. Krzyzańska A. McInnes A. Law I. J. Howieson J. (2005). European origin of *Bradyrhizobium* populations infecting lupins and *Serradella* in soils of Western Australia and South Africa. *Appl. Environ. Microbiol.* 71 7041–7052. 10.1128/AEM.71.11.7041-7052.2005 16269740 PMC1287703

[B58] Tamimi S. M. Young J. P. W. (2004). *Rhizobium etli* is the dominant common bean nodulating rhizobia in cultivated soils from different locations in Jordan. *Appl. Soil Ecol.* 26 193–200. 10.1016/j.apsoil.2004.01.003

[B59] Tang J. Bromfield E. S. P. Rodrigue N. Cloutier S. Tambong J. T. (2012). Microevolution of symbiotic *Bradyrhizobium* populations associated with soybeans in east North America. *Ecol. Evol.* 2 2943–2961. 10.1002/ece3.404 23301163 PMC3538991

[B60] Taurian T. Ibañez F. Fabra A. Aguilar O. M. (2006). Genetic diversity of rhizobia nodulating *Arachis hypogaea* L. in central Argentinean soils. *Plant Soil* 282 41–52. 10.1007/s11104-005-5314-5

[B61] Tong W. Li X. Huo Y. Zhang L. Cao Y. Wang E. (2018). Genomic insight into the taxonomy of rhizobium genospecies that nodulate *Phaseolus vulgaris*. *Syst. Appl. Microbiol.* 41 300–310. 10.1016/j.syapm.2018.03.001 29576402

[B62] Ulrich A. Zaspel I. (2000). Phylogenetic diversity of rhizobial strains nodulating *Robinia pseudoacacia* L. *Microbiology* 146 2997–3005. 10.1099/00221287-146-11-2997 11065378

[B63] Wang L. Cao Y. Wang E. T. Qiao Y. J. Jiao S. Liu Z. S. (2016). Biodiversity and biogeography of rhizobia associated with common bean (*Phaseolus vulgaris* L.) in Shaanxi Province. *Syst. Appl. Microbiol.* 39 211–219. 10.1016/j.syapm.2016.02.001 26966063

[B64] Wang R. Chang Y. L. Zheng W. T. Zhang D. Zhang X. X. Sui X. H. (2013). *Bradyrhizobium arachidis* sp. nov., isolated from effective nodules of *Arachis hypogaea* grown in China. *Syst. Appl. Microbiol.* 36 101–105. 10.1016/j.syapm.2012.10.009 23295123

[B65] Wei G. Chen W. Zhu W. Chen C. Young J. P. W. Bontemps C. (2009). Invasive *Robinia pseudoacacia* in China is nodulated by *Mesorhizobium* and *Sinorhizobium* species that share similar nodulation genes with native American symbionts. *FEMS Microbiol. Ecol.* 68 320–328. 10.1111/j.1574-6941.2009.00673.x 19416352

[B66] Weir B. S. Turner S. J. Silvester W. B. Park D. C. Young J. M. (2004). Unexpectedly diverse *Mesorhizobium* strains and *Rhizobium leguminosarum* nodulate native legume genera of New Zealand, while introduced legume weeds are nodulated by *Bradyrhizobium* species. *Appl. Environ. Microbiol.* 70 5980–5987. 10.1128/AEM.70.10.5980-5987.2004 15466541 PMC522066

[B67] Zinga M. K. Jaiswal S. K. Dakora F. D. (2017). Presence of diverse rhizobial communities responsible for nodulation of common bean (*Phaseolus vulgaris*) in South African and Mozambican soils. *FEMS Microbiol. Ecol.* 93 1–16. 10.1093/femsec/fiw236 27915286

[B68] Zohary D. Hopf M. (1973). Domestication of plants in the old World. *Science* 182 887–894. 10.1126/science.182.4115.887 17737521

[B69] Zurdo-Piñeiro J. L. García-Fraile P. Rivas R. Peix A. León-Barrios M. Willems A. (2009). Rhizobia from Lanzarote, the Canary islands, that nodulate *Phaseolus vulgaris* have characteristics in common with *Sinorhizobium meliloti* isolates from mainland Spain. *Appl. Environ. Microbiol.* 75 2354–2359. 10.1128/AEM.02811-8 19218416 PMC2675199

