# Supplementary figures and images for: Phylogeography of the Bradyrhizobium spp. Associated With Peanut, Arachis hypogaea: Fellow Travelers or New Associations?
Source: Front Microbiol. 2019 Sep 4;10:2041. doi: 10.3389/fmicb.2019.02041 (PMC6737463; doi:10.3389/fmicb.2019.02041)

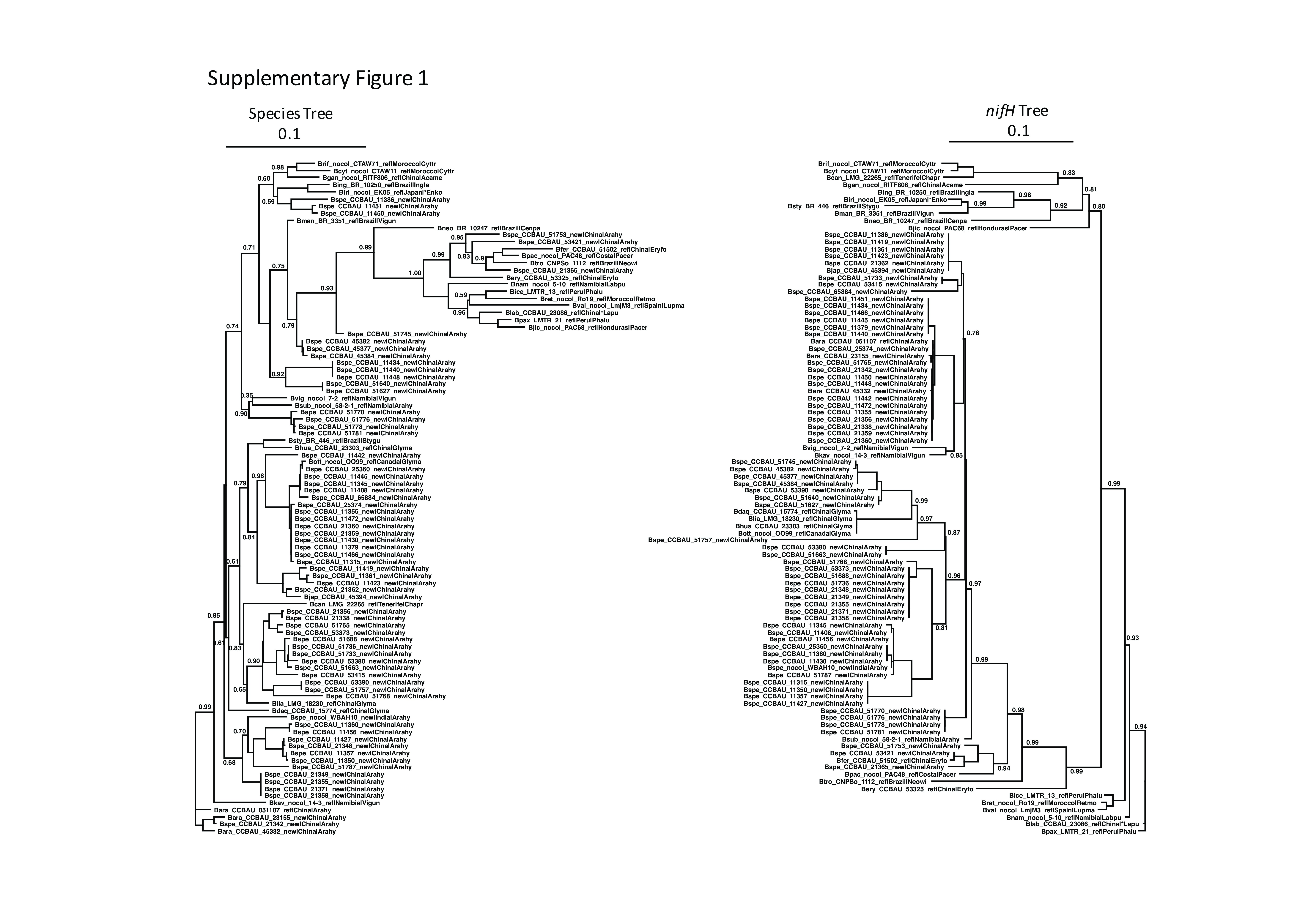

Supplement: FIGURE S1 — Presents the Maximum Likelihood trees, with branch lengths measured in substitutions per site, calculated under a GTR-Gamma model in RAxML, of the concatenated sequences of 16S region and the housekeeping genes recA, dnaK, glnII on the left, labeled “Species Tree”, and on the right, of the symbiotic nifH gene. [file Image_1.jpg]

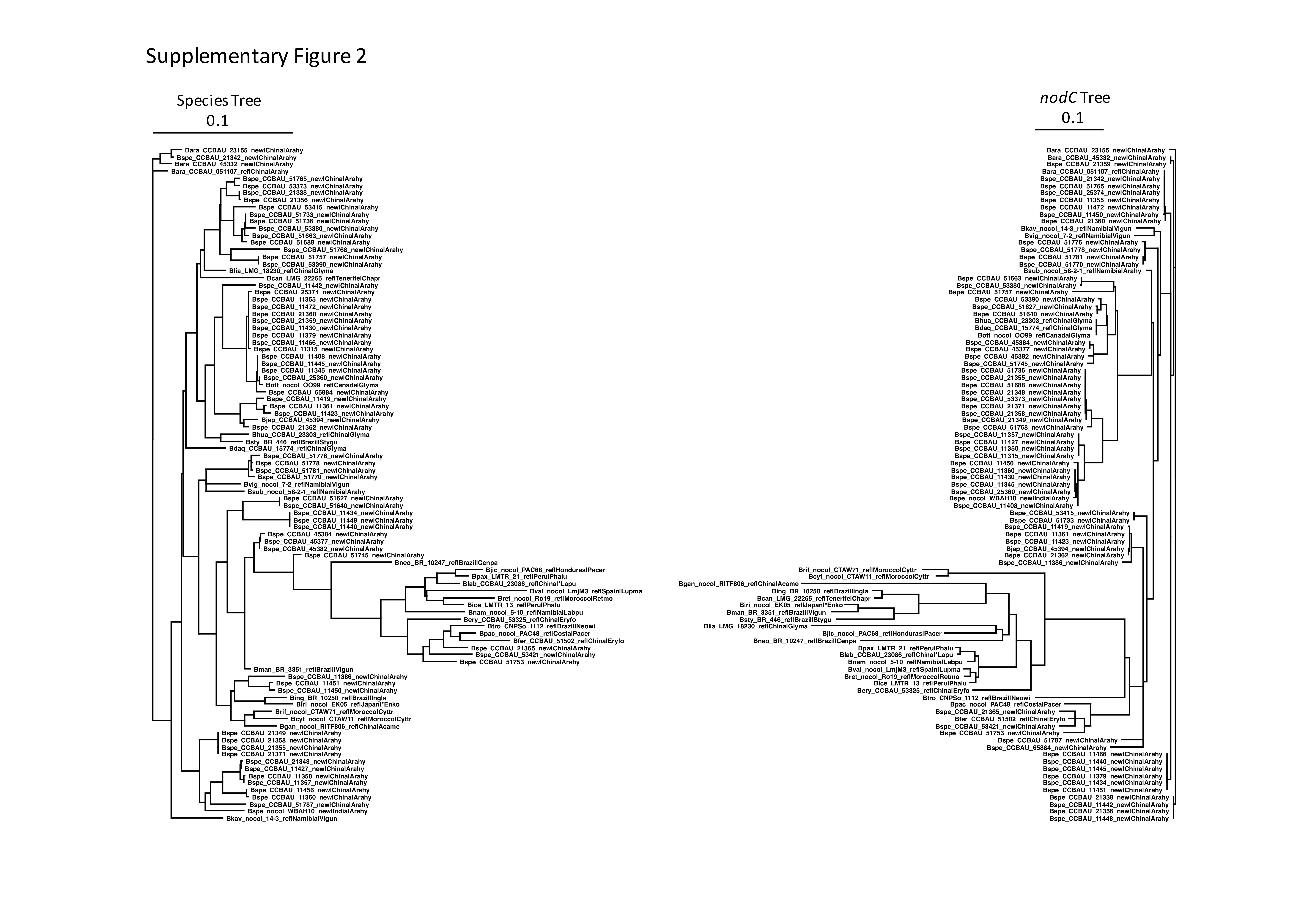

Supplement: FIGURE S2 — Presents the Maximum Likelihood trees, with branch lengths measured in substitutions per site, calculated under a GTR-Gamma model in RAxML, of the concatenated sequences of 16S region and the housekeeping genes recA, dnaK, glnII on the left, labeled “Species Tree”, and on the right, of the symbiotic nodC gene. [file Image_2.jpg]

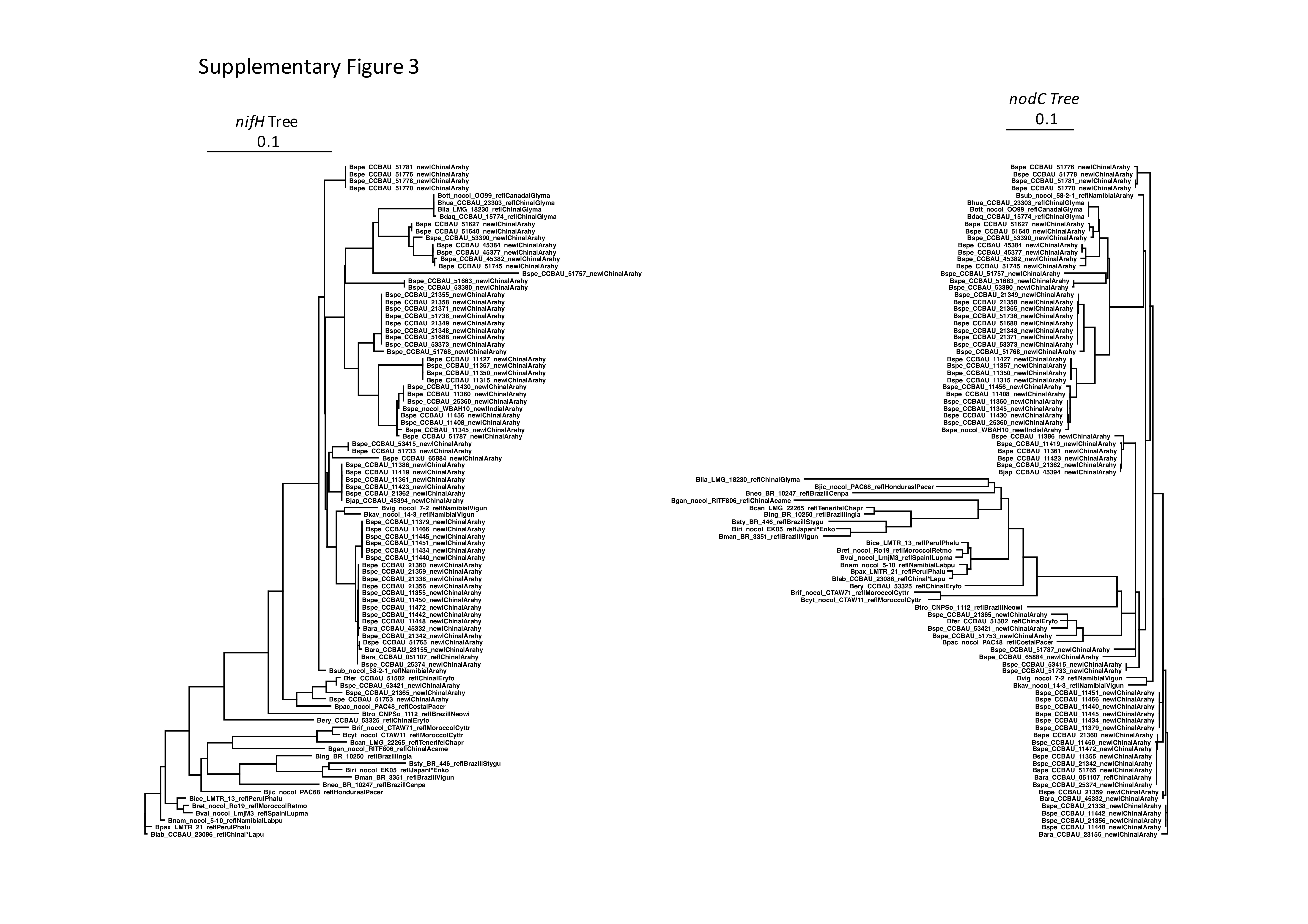

Supplement: FIGURE S3 — Presents the Maximum Likelihood trees, with branch lengths measured in substitutions per site, calculated under a GTR-Gamma model in RAxML, of nifH on the left and nodC on the right. [file Image_3.jpg]
